# Offspring-sex modifies the association between early-pregnancy adiposity and 2-year-old total physical activity – The Glowing Study

**DOI:** 10.21203/rs.3.rs-3179377/v1

**Published:** 2023-07-26

**Authors:** Eva Diaz, David Williams, E Howe, Elisabet Børsheim, Aline Andres

**Affiliations:** University of Arkansas for Medical Sciences; University of Arkansas for Medical Sciences; University of Arkansas for Medical Sciences; Arkansas Children’s Nutrition Center

**Keywords:** maternal programming, physical activity, adiposity, obesity, pregnancy

## Abstract

**Background:**

Rodent models suggest that *in utero* exposure to under and overnutrition programs offspring physical activity (PA) behaviors. Such nexus has not been established in humans. This study evaluated the association of early pregnancy maternal adiposity with offspring PA at age 2 years (2-yo-PA) taking into consideration prenatal and postnatal factors.

**Methods:**

Women (n=153) were enrolled early in pregnancy (<10 weeks). At enrollment, maternal adiposity [air displacement plethysmography, fat mass index (FMI, kg/m^2^)] and PA (accelerometers, activity counts) were measured, and age, race, and education self-reported. Gestational weight gain was measured at the research facility. Offspring birthweight and sex were self-reported. At age 2 years, parental feeding practices (child feeding questionnaire) were assessed, whereas anthropometrics (length and weight) and physical activity (accelerometers) were objectively measured. Offspring body mass index z-scores were calculated. Generalized linear regression analysis modeled the association of maternal FMI and 2-yo-PA [average activity counts (AC)^4^/day].

**Results:**

There was an interaction between maternal FMI and offspring sex in association with 2-yo-PA (β= −1.03, p= 0.030). Specifically, 2-yo-PA was lower in girls compared to boys when maternal FMI was ≥7 kg/m^2^. Maternal PA early in pregnancy positively associated with 2-yo-PA (β= 0.21, p= 0.005). In addition, children born to women with college education tended to be more active compared to children born to women without college education (β= 3.46, p= 0.059).

**Conclusions:**

Sexual dimorphism was observed in the associations of maternal adiposity with 2-yo-PA, with girls being less active compared to boys only when maternal FMI was ≥7 kg/m^2^.

## INTRODUCTION

The discovery of the developmental origins of health and disease, which emerged from studies of famine-exposed communities at the turn of the twentieth century (1, 2), has critical relevance in contemporary societies. In the United States, at least 50% of children are born every year to women with overweight [Body Mass Index (BMI) 25.0–29.9 kg/m^2^] or obesity (BMI ≥ 30 kg/m^2^) (3). Evidence supports that *in utero* exposure to maternal overweight/obesity increases cardiometabolic risk in the offspring, starting as early as birth and infancy. Maternal adiposity early in pregnancy, for example, is directly related to offspring adiposity at birth (4) and adiposity accrual in girls over the first two years of life (5). Indeed, by age two years, the obesity risk in children born to mothers with obesity is twice that of children born to women with normal weight (6). What is unknown is whether maternal obesity per se programs progeny behaviors pertinent to energy balance, such as physical activity behavior, thereby influencing children’s obesity risks..

A study in rodents reported that prenatal exposure to undernutrition (i.e., 50% of habitual calorie intake) and overnutrition (i.e., a high-fat [23%] diet) modified the disposition of progeny to engage in physical activity later in life (7). Specifically, compared to controls, exposed offspring exhibited markedly reduced degree of wheel-running activity. Similarly, using a mouse model, Johnson *et al.* (8) evaluated the effects of a high-fat diet during pregnancy on offspring physical activity. The authors reported offspring showed reduced voluntary physical activity when in the home-cage setting. In addition, compared to males, female offspring were more likely to sleep during dark cycles which are regarded as the time when rodents reach peak activity. These findings suggest that prenatal overnutrition may influence offspring’s attitudes and behaviors related to physical activity in a sex-dependent manner.

In humans, only one study has assessed the effects of gestational weight gain (GWG) on objectively measured physical activity in children (n = 113) (9). Physical activity was measured with accelerometers, and information on maternal pre-pregnancy weight status and GWG collected using recall questionnaires. At age 4 years, average activity counts per day decreased with increasing GWG in boys. In girls, the association between physical activity and GWG was modified by maternal overweight/obesity (n = 22) and followed an inverted U shape. These findings are consistent with animal models showing *in utero* overnutrition may program offspring’s physical activity patterns. The study is limited, however, by the small number of women with overweight and obesity as well as its cross-sectional design, which relied on maternal data recall.

Methodological difficulties likely account for the paucity of studies in humans assessing whether maternal overweight/obesity programs offspring physical activity behaviors. Separating the effects of prenatal and postnatal influences requires rigorous and costly studies with repeated measures over extended time periods. To address these research limitations, we conducted objective measurements of physical activity by accelerometry in 2-year-old participants of the longitudinal observational Growing Life, Optimizing Wellness study (GLOWING, NCT01131117). The GLOWING study’s primary goal is to assess the impact of maternal health before and throughout pregnancy on offspring obesity risk. On the basis of the limited evidence from murine models and human studies, we hypothesized that total physical activity (i.e., average daily activity counts) in 2-year-old offspring is inversely and sex-dependently associated with early-pregnancy maternal adiposity. Specifically, we hypothesized that physical activity declines with increasing maternal adiposity in 2-year-old boys and girls, but girls are significantly less active than boys.

## METHODS

### Subjects

Mother – offspring pairs (n = 153) were participants enrolled in the GLOWING study between 2011 and 2014 at the Arkansas Children’s Nutrition Center. The GLOWING study is an ongoing longitudinal observational study evaluating the impact of maternal health prior to and during pregnancy on offspring growth and obesity risk. Inclusion criteria were: BMI of 18.5–35 kg/m^2^ at enrollment, second parity, singleton pregnancy, ≥ 21 years old, and conception without assisted fertility treatments. Only offspring who were born full term, healthy and had no ongoing medical conditions at 2 years of age were eligible to participate in the present analysis. Exclusion criteria at enrollment and/or during pregnancy were: maternal preexisting or ongoing medical conditions including gestational diabetes, complications during pregnancy, medications known to influence fetal growth, maternal active smoking, alcohol consumption in any amount, and being an athlete (defined as being engaged in a professional sports activity). The institutional review board at the University of Arkansas for Medical Sciences approved the study protocol. All parents gave written informed consent.

### Measurements

#### Maternal measurements during pregnancy

##### Anthropometrics and gestational weight gain

Maternal height was measured to the nearest 0.1 cm at enrollment using a standard wall-mounted stadiometer (Tanita Corp., Tokyo, Japan). Body weight was measured at enrollment, 12-week gestation, and every 6 weeks thereafter using a tared scale (Perspective Enterprises, Portage, MI, USA) to the nearest 0.1 kg. Gestational weight gain was computed from the first measured weight to gestation week 36. Adherence to the Institute of Medicine (IOM) gestational weight gain guidelines was evaluated by adjusting the guidelines to reflect the last measure at gestation week 36 (10).

###### Maternal physical activity early in pregnancy

Average daily step counts and total activity counts were measured with the Actical accelerometer (Philips Respironics Co. Inc., Bend, Oregon, USA) at enrollment. The Actical is a small (2.8 × 2.7 × 1.0 cm; weight: 17g) accelerometer measuring omnidirectional gross motor activity. The device was placed on the participants’ ankles on the non-dominant side. Each participant wore the Actical for 2–5 days (2 days: n = 3; 3 days: n = 10; 4 days: n = 82; 5 days: n = 58). The monitor was programmed to record movement activity beginning at 11:59 PM on a given day. Participants were instructed to maintain usual activities while wearing the monitor. Total activity counts per day early in pregnancy were obtained using the visual identification method (refer to the ‘Offspring physical activity at age 2 years’ section for more details).

#### Demographic characteristics

Mothers self-reported race (i.e., American Indian or Alaska Native, Asian, Black or African American, Hispanic or Latino, Native Hawaiian or other Pacific Islander, or White), age, and date of last menstrual period. At the post-natal two-week research study visit, participants reported infant’s birth weight, infant’s race, and sex. Weight for gestational age at birth z-scores were computed from the maternal reported infant’s birth weight using the International Fetal and Newborn Growth Consortium for the 21st Century (INTERGROWTH – 21) standards (11).

#### Parental feeding practices at age 2 years

Parental feeding practices were measured using the Child Feeding Questionnaire (CFQ) (12). This scale targets parents of children ages 2 to 11 years and is comprised of 7 subscales. Four of them evaluate parental beliefs toward their child’s obesity proneness, and 3 evaluate parental control attitudes and practices regarding child feeding. The latter 3 were used in this study and include: restriction (limit access to certain foods), monitoring (keep track of what the child eats), and pressure to eat (pressure the child to eat more food, particularly during meals).

#### Offspring measurements at age 2 years

##### Anthropometric measurements at age 2 years

Offspring weight was measured to the nearest 0.01 kg using tared scale (SECA 727, SECA, Ontario, CA) and length was measured to the nearest 0.1 cm by using a length board (Easy Glide Bearing Infantometer, Perspective Enterprises, Portage, MI). BMI z-scores (BMI-Z) were computed based on the World Health Organization (WHO) Child Growth Standards for children ages 0 to 5 years (13).

#### Physical activity at age 2 years

Children wore Actical accelerometer at the ankle for 2 to 9 days (2 days: n = 4; 3 days: n = 7; 4 days: n = 5; ≥5 days: n = 137). The Actical data were downloaded using the Actical software. Data were summarised as 60-second epochs. Data in 60 second epochs were processed using a custom semi-automated algorithm to identify waking wear time (14). We adapted this algorithm (originally developed for uniaxial or triaxial accelerometer worn on the hip) with a refinement sample (n = 10), where rules were trialled and evaluated graphically, relative to visual identification. Several thresholds were trialled, and then the one informally judged to be the best performing (considering not only error amount, but the type of failures and how often they were likely to occur) was selected. Briefly, the algorithm attempts to identify in-bed and non-wear periods by searching for prolonged periods (≥ 180 minutes) of low activity (rolling averages of 30 minutes either side of each minute < 50cpm). The algorithm then searches for sustained periods of higher intensity activity (rolling average 30 minutes either side of ≥ 50 counts per minute for ≥ 10 minutes). A single non-wear rule was applied to all of the data. All minutes in continuous periods of ≥ 120 minutes of zero counts per minute, allowing for < 3 minutes with counts 1 to 50 counts per minute, were classed as non-wear. To screen out low-movement periods when the device was removed after data collection, any in-bed wear ≥ 24 hours after the last valid day was treated as invalid data for in-bed wear. Daily total activity counts and counts per minute were determined for waking wear periods as well as for rest periods. Participants were included in analyses if they had at least one day of 10 + hours of wear. Data were screened for abnormal values, and outliers were visually examined by two researchers to reach a consensus decision.

### Statistical analysis

Data measures in the interval scale are summarized as mean ± SD whereas data measures in the ordinal or nominal scale are summarized as percentages and counts. Categorical variables between groups were compared using the Chi-square or Fisher exact tests. A generalized linear model was used to model the association of 2-year-old physical activity (response variable) with early pregnancy FMI (primary term of interest) and with other prenatal and post-natal variables. Prenatal variables included maternal characteristics at enrollment [i.e., age, education (i.e., college vs. not college education) and physical activity (i.e., average daily activity counts)], as well as GWG (i.e., adequate, inadequate, and excessive). Postnatal variables were offspring sex, offspring race, offspring birthweight z-score, BMI-Z at age 2 years, and parental feeding practices (restriction, pressure, and monitoring).

The interaction between early pregnancy FMI and covariates were assessed. The base model with the study’s primary terms of interest produced a significant early pregnancy FMI × offspring sex interaction (β = −1.14, p = 0.020). No interactions were observed between maternal adiposity and any other of the considered variables. Our final model was created using principles of “Purposeful Selection” advocated by Hosmer *et al.* (15). Briefly, we evaluated several subsets among the important confounding variables in addition to the terms in the base model. The best subset of these terms was identified by observation of minimum model AIC statistic. The normality of the residuals was assessed by SAS Proc Univariate with the Shapiro-Wilk normality test. The test was non-significant which is consistent with no departure from normality in the residuals. Statistical analyses were conducted with SAS^®^ 9.4 (Cary, NC, U.S.A).

## RESULTS

### Study participants (Table 1)

Women were predominantly Caucasian (89%), and ≈ 30 years old at the time of enrollment. Seventy-three percent (n = 112) had college education and 54% (n = 83) excessive weight. Forty-six percent, 36%, and 18% of women had adequate, excessive, and inadequate GWG, respectively. The average gestational age at birth was ≈ 39 weeks and 58% of children were boys.

### Generalized linear regression analysis evaluating bivariate associations of offspring physical activity (activity counts 10^4^) at age 2 years with prenatal and postnatal variables of interest (Table 2)

Children born to Black mothers had an average of 67,300 less activity counts per day than those born to White mothers (β = −6.73, p = 0.025) (Table 2). Similarly, 2-year-olds born to college-educated mothers were more physically active than those born to non-college-educated mothers (β = 5.13, p = 0.008). Maternal physical activity (i.e., average activity counts per day × 10^4^) measured early in pregnancy positively associated with offspring total physical activity at 2 years of age (β = 0.20, p = 0.014). There was no association between 2-year-olds total physical activity and maternal age, maternal adiposity, GWG, birthweight z-score, and parental feeding practices (i.e., restriction, pressure, and monitoring).

### Generalized linear regression analysis: base and final models evaluating associations of 2-year-old offspring total physical activity (activity counts 10^4^) with prenatal and postnatal variables of interest (Table 3)

In the final model, maternal FMI, offspring sex, the interaction of maternal FMI with offspring sex, maternal physical activity, and maternal education were retained (Table 4). Compared to boys, the average activity counts per day of girls decreased with increasing maternal adiposity (**Figure**). The beta estimate of the final model for the maternal FMI × offspring sex interaction (β = −1.03, p = 0.031) changed by 9.6% from that obtained in the base model (β = −1.14, p = 0.020). Similarly, the beta estimate of the final model for early pregnancy physical activity (total activity counts × 10^4^) did not change compared to bivariate associations (bivariate association: β = 0.21, p = 0.014 vs. final model: β = 0.21, p = 0.005). For every 10,000 activity counts accrued early in pregnancy, 2-year-old offspring daily activity counts increased by 2,100. After accounting for the interaction of maternal adiposity × offspring sex and maternal physical activity early in pregnancy, the beta estimate for maternal education decreased by 30% compared to bivariate associations (bivariate association: β = 5.0, p = 0.012 vs. final model: β = 3.46, p = 0.059).

The association between 2-year-old offspring physical activity with sex was compared at different levels of maternal adiposity (FMI = 4, 5, 6, 7, 8, 9, and 10 kg/m^2^) (Table 4). In both adjusted and unadjusted models, offspring total physical activity at age 2 years was significantly lower in girls compared to boys when early pregnancy maternal FMI was ≥ 7 kg/m^2^ (Table 5).

### Generalized linear regression analysis evaluating the best fitted model for 2-year-old girls’ and boys’ physical activity (Table 5, Fig. 2).

**Figure 2:**
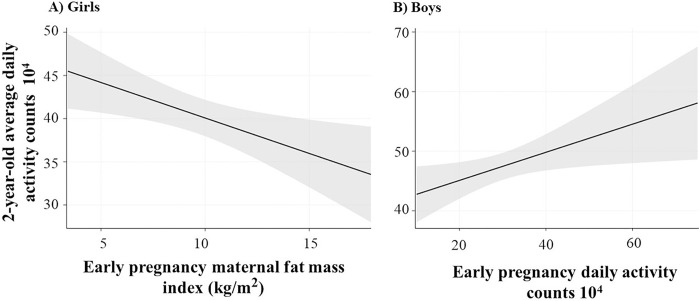
Fitted plot showing the association of 2-year-old offspring daily activity counts with early pregnancy maternal fat mass index (2A), and early pregnancy maternal daily activity counts (2B).

For girls, the best fitted model included early pregnancy adiposity (β = −0.66, p = 0.037) and maternal education (β = 4.69, p = 0.052) (Fig. 2A). Maternal education caused a 20% change in the β estimate of early pregnancy adiposity (from β = −0.82 to β = − 0.66) therefore it was retained in the final model. For boys (Fig. 2B), early pregnancy physical activity was the strongest predictive variable of offspring physical activity. Specifically, for every 10,000 activity counts early in pregnancy, 2-year-old boys’ daily activity counts increased by 2,400.

## DISCUSSION

This study aimed to examine the relationship between early pregnancy maternal adiposity and objectively assessed 2-year-old offspring physical activity, taking into account prenatal and postnatal factors that may impact this outcome. We specifically hypothesized that, offspring physical activity is inversely associated with early pregnancy maternal adiposity in both sexes, but girls are less active than boys. Our data showed an interaction between offspring sex and early pregnancy maternal adiposity in relation to 2-year-olds physical activity. Specifically, girls were less active compared to boys in mothers with early pregnancy FMI ≥ 7.0 kg/m^2^. In addition, maternal physical activity early in pregnancy and maternal education directly associated with total physical activity of 2-year-old offspring. When analyses were stratified by sex, early pregnancy maternal adiposity and maternal education were the strongest variables associated with girls’ physical activity. On the other hand, early pregnancy maternal physical activity was the strongest predictor of boy’s physical activity.

Little research has been conducted to study maternal obesity programming of offspring physical activity behaviors. Murine models have shed some light indicating that *in utero* exposure to undernutrition and overnutrition impacts adult offspring physical activity patterns in a sex-dependent manner, but results from these studies are contrasting. Cunha et al. (7) allocated pregnant Sprague-Dawley rats to receive one of three diets: standard chow (control, CTR), restricted diet (R) which delivered 50% of the average intake of the CTR group, and high-fat diet (HF) containing 23% fat. At birth, offspring were cross-fostered with other dams fed standard chow. Offspring were fed standard chow from weaning until completion of the study. Running wheel activity was measured for 7 consecutive days once offspring reached adulthood (postnatal day 60). Birthweights of R and HF groups were significantly lower compared to the CTR group. Male and female birthweights were comparable in the R and HF groups whereas males were heavier than females in the CTR group. Aside from birthweight, body weight did not differ between groups across the study. Adult male offspring from the HF group displayed lower wheel activity compared to CTR males. On the other hand, adult females from the HF group were more active compared to CTR females.

These results are in contrast with those reported by Johnson et al. (8). It is worth mentioning, however, that Johnson et al. used a biparental and monogamous oldfield mouse model. In addition, offspring were fostered with both biological parents until weaning, at which point they were fed standard chow. Dams were fed either a CTR or HF diet starting 2 weeks prior to mating and throughout the experiment. Anxiety behavior was assessed using the Elevated Plus Maze (EPM). HF female offspring showed decreased mobility in the EPM indicating they were more anxious than CTR females. Wheel running activity did not differ between the CTR and HF groups; however, HF females walked less and had lower resting energy expenditure during dark cycles compared to CTR females.

Taken together, these rodent models suggest that adult physical activity behaviors are programed during prenatal development. Differences in rodent models, and age of testing may explain the variation in results between studies. What remains unknow is how physical activity behaviors in younger offspring relate to maternal obesity in pregnancy. Our data show that the association of 2-year-old offspring total physical activity with early pregnancy maternal adiposity is modified by offspring sex. Girls were less active than boys when maternal adiposity early in pregnancy was ≥ 7 kg/m^2^. Sexual dimorphism in physical activity has been reported in children and adolescents (4 to 18 years) with girls spending more time in sedentary activities compared to boys (16–18). It is less clear whether physical activity in infancy and toddlerhood (2 to 3 years of age) varies by sex. Hager et al. (19) evaluated physical activity in toddlers from low-income families (n = 191) from the Baltimore, Maryland region in the United States. Both, mother and offspring wore Actical accelerometers. In this study, 73% of children were < 2 years of age, mothers were predominantly Black (68%), had overweight/obesity (72%), and completed high school education only (72%). Male sex, non-Black race, and maternal physical activity were all directly associated with offspring moderate to vigorous physical activity.

While the primary outcome of the aforementioned study and ours are different, the results of Hager et al. are pertinent to our study. Sex differences in infants’/toddlers’ physical activity levels were observed in a population primarily comprised of Black women with excessive weight. Other studies objectively measuring physical activity levels of infants/toddlers do not report differences in physical activity between boys and girls. For instance, Hnatiuk et al. (20) characterized physical activity levels and patterns of 18 to 19 month-old Australian children (n = 295) from the Melbourne region. Physical activity was measured using hip-worn GT1M (uniaxial) Actigraph accelerometers. In this study, total daily physical activity, and minutes in moderate to vigorous physical activity did not differ between boys vs. girls (delta = 3.6 minutes/day), although, boys were more active than girls in the morning hours. This study did not report on maternal weight status and 75% of women had post-secondary education. Bivariate associations from our study (Table 2) showed that female offspring and Black maternal race negatively associated with offspring total physical activity. However, when maternal adiposity was considered, sex-differences in offspring born to women with FMI < 7 kg/m^2^ were attenuated and became evident when this FMI cutoff was exceeded. Contrary to Hager’s study, our participants were predominantly educated Caucasians (89%), so it is possible that a lack of statistical power prevented us from conclusively linking maternal Black race to offspring physical activity in our final model. Whether the association with maternal race is driven by social disparities or biology, cannot be established with the current study design. Our data suggests the possibility that prenatal exposure to maternal overweight/obesity programs early life offspring physical activity behaviors in a sex dependent manner. However, more studies are needed to confirm this finding. We have previously reported that dietary palmitate (i.e., fat) oxidation is lower in 2-year-old girls than in boys, and that early pregnancy maternal adiposity directly associates with girls’ but not boys’ adiposity accrual over the first two years of life (5, 21). It is unknown if sexual dimorphism in offspring physical activity behaviors is contributing to these findings.

Wasenius et al. (9) found a negative association between self-reported maternal gestational weight gain (kg) and objectively measured physical activity (accelerometry) in 3.6-year-old children (β = −3.2, p = 0.049). When analyses were stratified by sex, they found an interaction between self-reported pre-pregnancy BMI status and gestational weight gain in association with offspring total physical activity. In girls but not boys, physical activity decreased with increasing gestational weight but only in offspring born to mothers with overweight or obesity (i.e., BMI ≥ 25 kg/m^2^). On the other hand, in women with normal weight, gestational weight gain directly associated with offspring physical activity. In our study, gestational weight gain (absolute [kg] or status [inadequate, adequate, excessive], did not associate nor interacted with maternal adiposity (or BMI) in relation to 2-year-old offspring physical activity. A limitation of the former study was that questionnaires were used to estimate gestational weight gain which could have increased recall bias.

Our study had some limitations including a sample comprised of mostly Caucasian mothers (89%) and living in Arkansas, which may limit the generalizability of the current findings to other racial or ethnic groups. However, the study also had numerous strengths including longitudinal collection of data, measurement of maternal anthropometrics directly in clinic, consideration of parenting styles, and most importantly direct objective measures of maternal and offspring physical activity using accelerometry.

## Conclusions

Two-year-old total physical activity decreased in girls compared to boys but only when early-pregnancy maternal adiposity exceeded 7 kg/m^2^. Early-pregnancy maternal physical activity directly associated with offspring physical activity, particularly in males. Maternal low socioeconomic status, as indicated by maternal education, inversely associated with offspring physical activity. In agreement with animal models, we observed sexual dimorphism in the associations of maternal adiposity during pregnancy and offspring physical activity behaviors with girls being less active compared to boys.

## Figures and Tables

**Figure 1:**
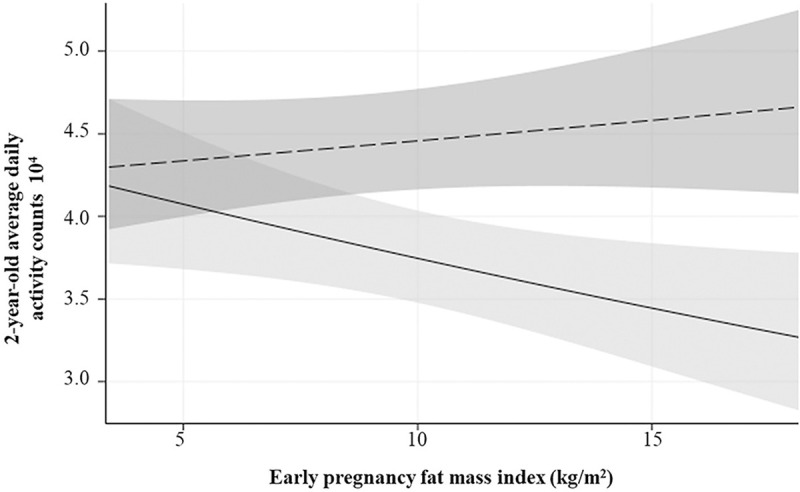
Regression plot showing the interaction between early pregnancy maternal adiposity (X axis) and offspring sex in association with 2-year-olds mean activity counts per day (Y axis).

**Table 1:** Participant characteristics

Variables	(n = 153)
Maternal age (years)	29.57 ± 3.53
Maternal race, n (%)	
White	136 (89)
Black	14 (9)
Other	3 (2)
Maternal education level, n (%)	
College degree	112 (73)
Not College degree	41 (27)
Maternal BMI at enrollment	25.88 ± 4.28
Maternal weight status at enrollment, n (%)	
Normal weight (BMI < 25 kg/m^2^)	70 (46)
Excessive weight (BMI ≥ 25 to < 35 kg/m^2^)	83 (54)
Maternal fat mass index at enrollment, (kg/m^2^) (n = 152)	9.46 ± 3.39
Early pregnancy maternal daily counts 10^4^	30.5 ± 10.68
Gestational weight gain, (kg) (n = 151)	11.96 ± 3.89
Gestational weight gain status, n (%)	
Adequate	70 (46)
Excessive	55 (36)
Inadequate	28 (18)
Gestational age at birth (weeks)	39.4 ± 1.13
Child sex, n (%)	
Boys	88 (58)
Girls	65 (42)
Length at birth (cm)	51.08 ± 2.61
Length at birth z-score	1.09 ± 1.36
Length at birth percentile	75.57 ± 29.83
Weight at birth, (kg)	3.48 ± 0.45
Weight z-score at birth	0.57 ± 1.00
Weight percentile at birth	66.4 ± 27.67
BMI z-score at age 2 years	0.4 ± 1.01
Offspring daily activity counts 10^4^ at age 2 years	44.5 ± 10.94
Parental feeding practices at age 2 years	
Restriction	26.24 ± 5.48
Pressure	9.08 ± 3.5
Monitoring	11.36 ± 2.86

Data presented as mean ± SD, counts and percentages. BMI = body mass index. Unless specified otherwise the sample size for each variable is n = 153

**Table 2:** Bivariate associations

Variable	β	SE	95% CL		p-value
Maternal age (years)	0.27	0.25	−0.22	0.76	0.2823
Maternal race					
Other	0.75	6.26	−11.52	13.03	0.9043
Black	−6.73	3.01	−12.64	−0.83	**0.0254**
White (reference)					
Maternal education					
College education	5.13	1.95	1.31	8.95	**0.0084**
Not-college education (reference)	0.00	0.00	0.00	0.00	.
Maternal fat mass index (kg/m^2^)	−0.22	0.26	−0.73	0.29	0.3980
Gestational weight gain (kg)	−0.03	0.23	−0.48	0.42	0.9124
Weight-for-sex and gestational age at birth z-score	−0.79	0.89	−2.53	0.95	0.3719
Parental feeding practices					
Restriction	−0.07	0.16	−0.39	0.24	0.6497
Pressure	−0.36	0.25	−0.85	0.13	0.1530
Monitoring	−0.43	0.31	−1.03	0.17	0.1618
Early pregnancy physical activity (AC × 10^4^)	0.20	0.08	0.04	0.36	**0.0135**

SE = standard error, CL = confidence limits

**Table 3:** Best fitted model for total physical activity in 2-year-old children

Parameter	β	SE	95% CL		p-value
Maternal FMI	0.40	0.32	−0.22	1.02	0.2008
Child sex Female	2.46	4.80	−6.94	11.87	0.6080
Male (reference)					
Maternal FMI × child Female	−1.03	0.48	−1.97	−0.10	**0.0307**
Male (reference)					
Maternal physical activity count × 10^4^	0.21	0.07	0.06	0.36	**0.0054**
Maternal education College degree	3.46	1.84	−0.13	7.06	**0.0592**
Non-college degree					

SE = standard error, CL = confidence limit, FMI = fat mass index

**Table 4:** Two-year-old’s physical activity at different levels of early pregnancy maternal adiposity

Early Pregnancy Maternal Fat Mass Index (FMI)	Mean β	Mean β 95% CI		χ 2	p-value
**Unadjusted associations**					
FMI = 4	−0.93	−7.12	5.25	0.09	0.7680
FMI = 5	−2.07	−7.46	3.32	0.56	0.4523
FMI = 6	−3.21	−7.87	1.46	1.81	0.1780
FMI = 7	−4.34	−8.37	−0.31	4.46	**0.0348**
FMI = 8	−5.48	−9.03	−1.93	9.15	**0.0025**
FMI = 9	−6.62	−9.90	−3.33	15.59	**< 0.0001**
FMI = 10	−7.75	−11.04	−4.47	21.36	**< 0.0001**
**Adjusted associations**					
FMI = 4	−1.67	−7.67	4.33	0.30	0.5849
FMI = 5	−2.71	−7.93	2.52	1.03	0.3100
FMI = 6	−3.74	−8.25	0.77	2.64	0.1040
FMI = 7	−4.77	−8.66	−0.88	5.79	**0.0161**
FMI = 8	−5.81	−9.22	−2.39	11.09	**0.0009**
FMI = 9	−6.84	−10.00	−3.68	18.00	**< 0.0001**
FMI = 10	−7.87	−11.04	−4.70	23.68	**< 0.0001**

Estimates (β) are presented on the scale of the response variable. CI = confidence interval, χ2 = chi square. Adjusted associations: estimates adjusted for early pregnancy maternal physical activity and maternal education.

**Table 5:** Best fitted model for girls and boys

Parameter	β	SE	95% CL		p-value
**Girls**					
Early pregnancy FMI	−0.66	0.32	−1.27	−0.04	0.0370
Maternal education					
College education	4.69	2.41	−0.04	9.42	0.0522
Non-college education (reference)					
**Boys**					
Early pregnancy physical activity (activity counts 10^4^/day)	0.24	0.10	0.03	0.44	0.0239

FMI = fat mass index, SE = standard error, CL = confidence limits

## Abbreviations

BMI: Body mass index
CTR: Control
EPM: Elevated Plus Maze
FMI: Fat mass index
GWG: Gestational weight gain
HF: High fat
R: Restricted
